# The Effect of Honeycomb-Structured Hydrophilic–Hydrophobic Mixed Surfaces on the Spreading Process of Liquid Droplets

**DOI:** 10.3390/biomimetics10040209

**Published:** 2025-03-28

**Authors:** Chenyue Zhu, Mark Alston, Yuying Yan

**Affiliations:** Faculty of Engineering, University of Nottingham, University Park, Nottingham NG7 2RD, UK; laxcz17@exmail.nottingham.ac.uk (C.Z.); mark.alston@nottingham.ac.uk (M.A.)

**Keywords:** honeycomb structure, molecular dynamics simulation, surface wettability

## Abstract

Honeycomb-structured, mixed-wettability surfaces have attracted significant attention due to their potential for tailoring surface properties and controlling fluid dynamics at the nanoscale. However, the underlying mechanisms governing droplet spreading and wettability modulation remain insufficiently understood. This study, using molecular dynamics simulations, reveals that periodic hydrophilic–hydrophobic areas within honeycomb structures induce unique oscillatory spreading behaviors and allow the precise modulation of equilibrium contact angles. The findings demonstrate that honeycomb designs can effectively transition surfaces between hydrophilic and hydrophobic states, with practical applications in boiling heat transfer, thermal management, and advanced materials development.

## 1. Introduction

The spreading of liquid droplets on a solid substrate is critical in nature and in various industries, with extensive applications in lubrication, painting, surface coating, oil recovery, and so forth. In the 1970s, researchers from the US and Japan began to process micro/nano-structured patterns on boiling surfaces to improve the boiling heat transfer performance of electronic device liquid cooling technology [1]. Emerging techniques by micro/nano-fabrication technology to form surfaces have gradually shifted from the conventional scale to the micro/nanoscale. With the development of MEMS and NEMS devices, this reduction in scale has continued, while the power density of such technology has increased [2]. This trend has led to a significant rise in the heat generated per unit area, making effective thermal management increasingly challenging. Boiling heat transfer is a highly efficient method, acting as a heat exchange mechanism, which has been proven by previous key experimental research.

It has been demonstrated that a mixed-wettability surface can effectively enhance boiling heat transfer. Hu et al. [3] observed that hydrophobic surfaces exhibit better boiling heat transfer coefficients compared to hydrophilic surfaces, although the critical heat flux (CHF) is lower. Zhang et al. [4] found that under low superheat conditions, hydrophobic surfaces have a lower boiling onset temperature and higher heat transfer coefficients than hydrophilic surfaces. In addition, researchers have employed the lattice Boltzmann method to investigate the impact of surface wettability on boiling at the microscale [5]. Lee et al. [6] demonstrated that reducing the wettability can increase the heat transfer coefficients, but decreases the CHF. Jo et al. [7] studied bubble nucleation sites, detachment frequency, and other factors of hydrophobic and hydrophilic surfaces in regard to nucleate boiling.

Experimental work underpins nature-inspired structures that have resulted in enhanced thermal exchange of the surface in comparison to traditional surfaces. The unique wettability properties found in nature have been used with multi-scale morphological structures found on the surfaces of various plants and animals to analyze aspects such as the “lotus effect” [8]. In 1997, German botanists Barthlott and Neinhuis discovered that the surface of lotus leaves exhibits distinctive micron-scale papillae structures, allowing water droplets to roll freely across the surface and carry away surface contaminants, thereby achieving a self-cleaning effect. A similar phenomenon of unidirectional liquid transport has been observed on the surface of rice leaves. The surface of rice leaves features micro–nano composite protrusions like those of lotus leaves. These unevenly arranged protrusions are the primary cause of the isotropic rolling behavior of water droplets on the surface of the leaves [9]. In addition to lotus leaves, the wettability phenomenon of rice leaves and roses [10,11] can also be observed in other plants. Moreover, the wettability of insect wing surfaces has garnered significant attention in recent studies.

The investigation into honeycomb structures generated by nature as a thermal exchange surface is limited. Honeycomb structure refers to highly ordered micro/nanoscale structures arranged in a hexagonal pattern. This geometry of highly uniform and regular hierarchical structures can theoretically serve as model for studying thermal properties to generate a surface pattern [12,13,14]. The arrangement of structured formations in regard to a specific surface area can be used to form unique optical properties, making them promising candidates for applications in various fields, including tissue engineering, life sciences, superhydrophobic materials, optoelectronic materials, templates, sensors, and micro/nanoparticle separation [15,16,17,18,19,20,21,22,23,24,25,26,27,28,29].

As a result, in this research, we employ the honeycomb structure to create a new mixed hydrophilic and hydrophobic surface to observe how a mixed-wettability structure with a honeycomb structure effects the wettability of the surface and how droplets spread on these surfaces to add further insight into the enhancement of boiling heat transfer.

However, at the nanoscale, it is challenging to observe bubble nucleation or measure the heat flux density through the use of experimental methods or traditional macroscale numerical simulation approaches. Due to the limitations in terms of scale, traditional experimental and numerical simulation methods are unable to effectively reveal the mechanisms of phase change. Molecular dynamics (MD) has emerged in recent years as a powerful microscale numerical simulation method. This method is based on Newton’s second law to simulate molecular motion, enabling the analysis of the microscopic evolution of molecular systems. By applying statistical methods, the macroscopic thermodynamic properties of the molecular system can be calculated.

With advancements in computational modeling, MD has been widely adopted and promoted, becoming a robust tool for exploring the mechanisms of boiling heat transfer. Zhang et al. [30] demonstrated through molecular dynamics (MD) simulations that surface wettability is one of the primary factors influencing boiling heat transfer. A study by Wang et al. [31] concludes that enhanced surface wettability reduces interfacial thermal resistance, thereby promoting boiling heat transfer. Similarly, Cao and Cui [32] found that the lower vibrational density of states (VDOS) mismatch between hydrophilic solid walls and liquids results in better heat transfer performance of the liquid film on hydrophilic surfaces. In addition, Hens et al. [33] showed that hydrophilic surfaces, due to their stronger solid–liquid interfacial potential, can significantly improve boiling heat transfer performance. Chen et al. [34] observed that during boiling, a layer of argon atoms consistently adheres to hydrophilic triangular nanochannels. This work concludes that hydrophilic nanochannels have greater capacity to transfer thermal energy than hydrophobic surfaces, thus promoting bubble nucleation. Yin et al. [35], by analyzing the potential energy distribution of liquid films, demonstrated that enhanced surface wettability increases bubble nucleation rates. Wang and Li [36] revealed that hydrophilic–hydrophobic mixed surfaces with a high hydrophilic area ratio exhibit lower interfacial thermal resistance and superior boiling heat transfer performance.

In this study, molecular dynamics simulation is employed to further investigate the role of the honeycomb structure in modifying surface wettability. Specifically, the spreading process of liquid water molecules at room temperature on a honeycomb-etched mixed-wettability surface is simulated. We adjust the wettability of both the honeycomb-etched surface and the smooth surface by tuning the solid–liquid interactions to create a mixed hydrophilic–hydrophobic surface. This study examines the effects of the honeycomb-etched structure on surface wettability, the evolution of the water droplet spreading radius R, and how the honeycomb structure influences the motion of water molecules. Additionally, the effect of the etched honeycomb structure on surfaces with different wettability is also investigated.

**Figure 1:**
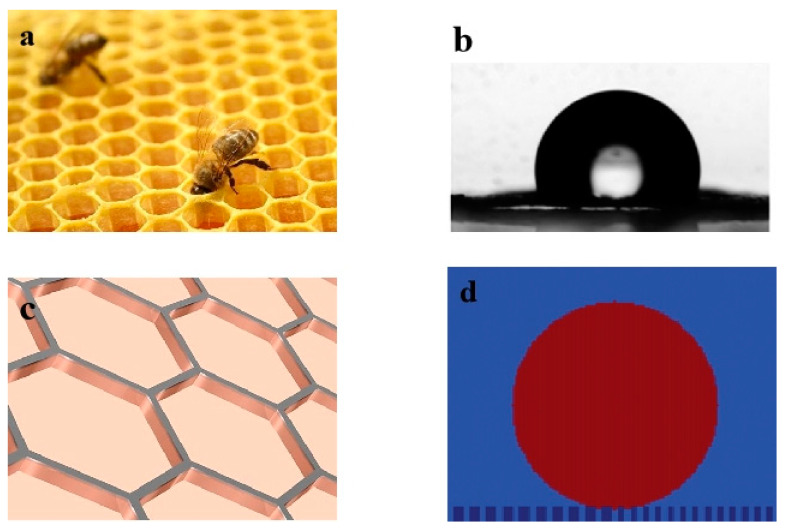
(**a**) A photograph of a honeycomb structure found in nature; (**b**) a photograph of the water droplet shape on the surface of the honeycomb structure [37]; (**c**) the applied geometry of the honeycomb structure to a smooth surface; (**d**) molecular dynamics simulation model of water droplet spreading on a honeycomb-etched structure surface.

Figure 1a illustrates a natural honeycomb structure with a highly uniform hexagonal array pattern. Figure 1b shows the hydrophobic state of water droplets on the cell walls of the natural honeycomb surface under a microscope. Guo et al. [37] discovered that the wettability and adhesion properties of natural honeycomb walls in regard to water and honey droplets can be utilized as a “mechanical hand” for micro-droplet transfers. Zhang et al. [2] progressed this work through the analysis of transported micro/nano droplets by modifying the surface wettability through stretching the honeycomb structure pores. To gain deeper insights into the role of honeycomb walls in regard to surface wettability for micro-droplet transfers, we created a honeycomb form by etching this structure onto a smooth surface, as highlighted in Figure 1c. The molecular simulation effects of the applied honeycomb-structured pattern was assessed by modelling the surface geometry roughness effects, as illustrated in Figure 1d, to understand its influence on surface wettability.

## 2. Methodology

**Figure 2:**
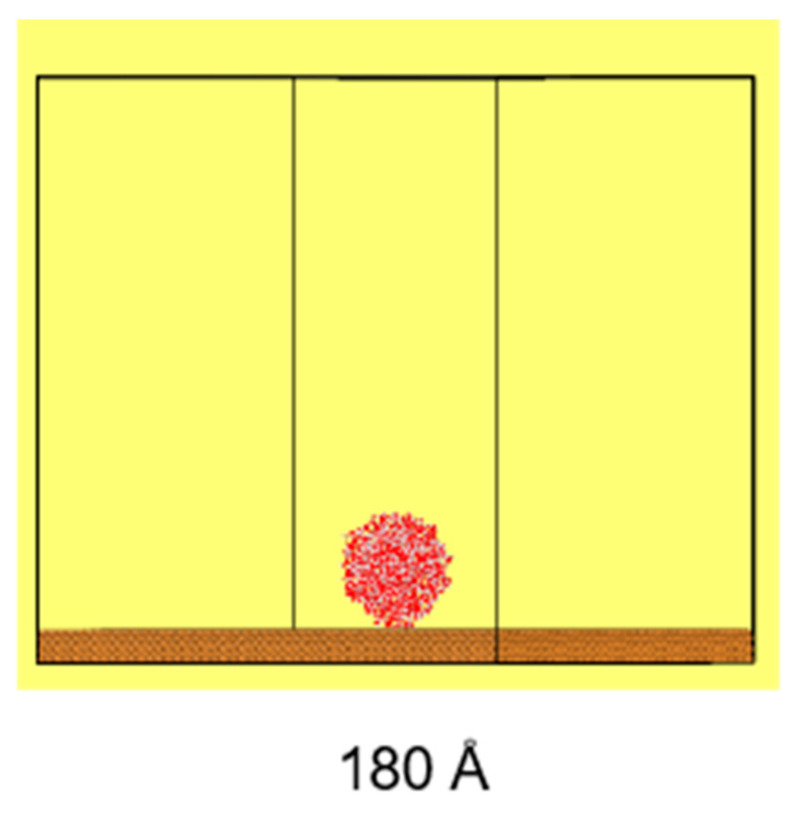
Schematic diagram of the simulation model.

The simulation system includes a cylindrical water droplet, with a diameter of 100 Å and a length of 22 Å, containing 2000 water molecules, maintaining a bulk water density of approximately 1 g/cm^3^. This droplet is placed onto a copper (Cu) substrate, featuring etched hexagonal structures, with a lattice constant of 3.61 Å, as illustrated in Figure 2. The use of cylindrical water droplets in simulations offers computational advantages over spherical droplets, as it enhances the computational efficiency and eliminates the line tension effect. Additionally, as demonstrated by Heine et al. [38], the behavior observed in simulations with cylindrical droplets is consistent with that of spherical droplets, validating the approach for studying interfacial phenomena.

The size of the substrate is 180 Å × 180 Å × 50 Å (X × Y × Z), while the size of the simulation domain is set at 180 Å × 180 Å × 200 Å (X × Y × Z). The simulation domain in the Z direction is set to avoid non-physical interactions and phenomena caused by the simulation cycle, which ensures that the droplet above is not affected by the substrate.

**Figure 3:**
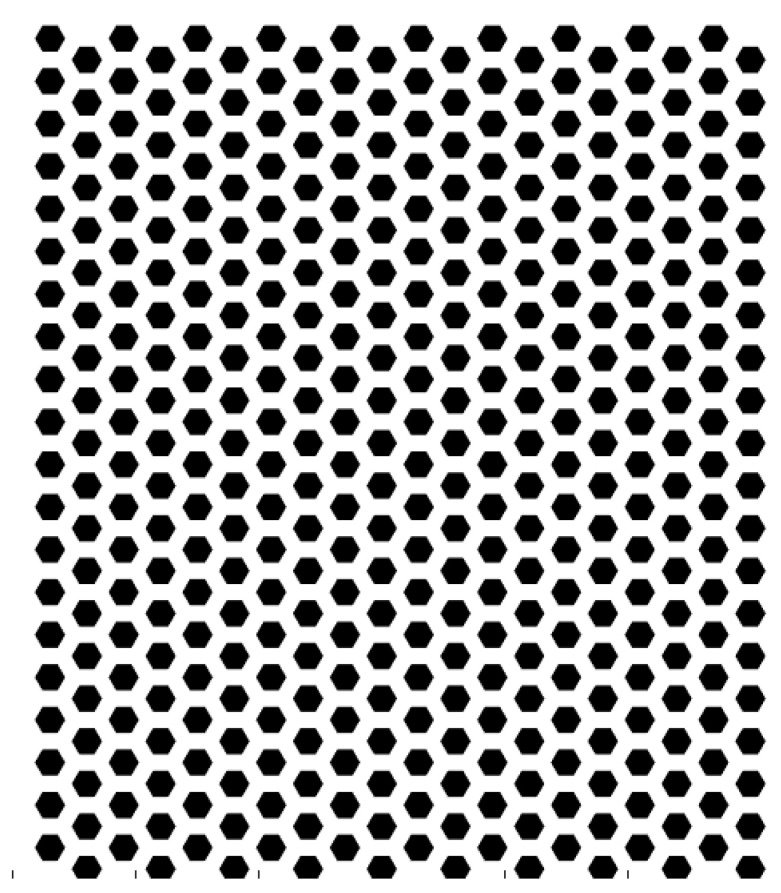
Etched honeycomb structure in 2D (solid black areas illustrate etching pattern).

The etched honeycomb structure is designed as shown in Figure 3. The hexagonal honeycomb structure has a unit size of 9 Å (angstroms), with 20 units evenly distributed along both the X and Y directions on the substrate. The etching depth of the area is 1.8 Å.

To achieve a balance between computational efficiency and accuracy, the extended simple point charge (SPC/E) water model is employed. This model accounts for Coulombic (electrostatic) interactions, van der Waals interactions, and bonded interactions, including bond stretching and angle bending terms, as described in Equations (1)–(4). This approach ensures reliable simulation results, while optimizing the computational resources.

(1) $$U_{\mathrm{Coul}}=\frac{e^{2}}{4\pi \varepsilon_{0}}\underset{i\neq j}{\sum }\frac{q_{i}q_{j}}{r_{\mathrm{ij}}}$$

(2) $$U_{\mathrm{VDW}}=\underset{i\neq j}{\sum }4\varepsilon_{\mathrm{ij}}\left[{\left(\frac{\sigma_{\mathrm{ij}}}{r_{\Gamma_{j}}}\right)}^{12}-{\left(\frac{\sigma_{\mathrm{ij}}}{r_{\Gamma_{j}}}\right)}^{6}\right]$$

(3) $$U_{\mathrm{bond}\;\mathrm{strech}}=\underset{\mathrm{Bonds}}{\sum }k_{1}{\left(l_{\mathrm{ij}}-l_{0}\right)}^{2}$$

(4) $$U_{\mathrm{angle}\;\mathrm{bend}}=\underset{\mathrm{Angles}}{\sum }k_{2}{\left(\theta_{\mathrm{ijk}}-\theta_{0}\right)}^{2}$$

In the equations, $U_{\mathrm{Coul}}$, $U_{\mathrm{VDW}}$, $U_{\mathrm{bond}\;\mathrm{stretch}}$, and $U_{\mathrm{angle}\;\mathrm{bend}}$ represent the potential energy contributions from Coulombic (electrostatic) interactions, van der Waals interactions, bond stretching, and angle bending, respectively. In Equations (1) and (2), $r_{\mathrm{ij}}$ denotes the distance between two atoms, e is the electron charge, $\varepsilon_{0}$ is the vacuum permittivity, and $q_{i}$ and $q_{j}$ are the partial charges of atoms i and j, respectively. The parameters $\varepsilon_{\mathrm{ij}}$ (depth of the potential well) and $\sigma_{\mathrm{ij}}$ (collision diameter) define the 12-6 Lennard-Jones (LJ) potential. In Equation (3), $k_{1}$ is the force constant, $l_{\mathrm{ij}}$ is the instantaneous O−H bond length, and $l_{0}$ is the equilibrium O−H bond length. Similarly, in Equation (4), $k_{2}$ is the force constant, $\theta_{\mathrm{ijk}}$ is the instantaneous H−O−H bond angle, and $\theta_{0}$ is the equilibrium H−O−H bond angle. For the SPC/E water model, the force constants $k_{1}$ and $k_{2}$ are 554.1349 kcal/(mol·Å^2^) and 45.7696 kcal/(mol·rad^2^), respectively, the equilibrium O−H bond length $l_{0}$ is 1 Å, and the equilibrium H−O−H bond angle is 109.47°.

Interactions between water molecules and solid atoms are described by the 12-6 LJ potential. Heine et al. parameterized the 12-6 LJ potential for several face-centered cubic (fcc) metals, including Al, Au, Pb, and Cu. The interaction parameter $\sigma_{O\text{-}S}$ is determined using the arithmetic mean rule [38]:

$$\sigma_{O\text{-}S}=\frac{\sigma_{O}+\sigma_{S}}{2}$$

where $\sigma_{O\text{-}S}$ ranges from 2.7195 to 3.1708 Å. The parameter $\varepsilon_{O\text{-}S}$ is calculated using the geometric mean rule:

$$\varepsilon_{O\text{-}S}=\sqrt{\varepsilon_{O}\times \varepsilon_{S}},$$

with values ranging from 0.6748 to 1.1010 kcal/mol. Here, the subscripts O and S refer to oxygen atoms and substrate solid atoms, respectively.

In this work, to explore different wetting behaviors, a wider range of $\varepsilon_{O\text{-}S}$ values (1 × $\varepsilon_{O\text{-}O}$ to 10 × $\varepsilon_{O\text{-}O}$, or 0.1554–1.554 kcal/mol) is set for the smooth substrate, while the etched area parameters remain fixed. For the copper (Cu) substrate, $\sigma_{O\text{-}S}$ is fixed at 2.8907 Å, and $\varepsilon_{O\text{-}S}$ is varied to investigate the influence of the potential well depth. Table 1 and Table 2 summarize the non-bonded potential parameters used in this study.

Table 2 indicates 6 simulation cases involving the etched structure to maintain the wettability-to-time duration in comparison to a smooth plate surface to ascertain the different wettability effects of the spreading phenomenon.

All the simulations were conducted in a canonical ensemble (NVT), with the temperature set to 300 K, controlled by a Nosé–Hoover thermostat. Periodic boundary conditions were applied in the X, Y, and Z directions to eliminate boundary effects. With periodic boundary conditions, the droplet can be considered infinitely long in the X direction, effectively forming a cylindrical droplet. During the spreading process, the three-phase contact line of the cylindrical droplet remains straight, thereby eliminating the influence of line tension caused by the curvature of the contact line on the contact angle. During the simulations, long-range Coulombic interactions were calculated using the Particle–Particle/Particle–Mesh (PPPM) method, with an accuracy of 99.99%, and the cutoff radius for the LJ 12-6 potential was set to 1 nm. To enhance the computational efficiency, the SHAKE algorithm embedded in LAMMPS was employed to constrain the O-H bonds and H-O-H angles of the water molecules, while the solid substrate remained fixed throughout the simulations. The equations of motion for the atoms were integrated using the leapfrog algorithm, with a time step of 1 fs. All the molecular dynamics (MD) simulations in this study were performed using the open-source software LAMMPS (LAMMPS 64-bit 27Oct2021), and the atomic trajectory files generated during the simulations were visualized using the open-source software OVITO 3.11.0. At the beginning of the simulation, the droplet, pre-equilibrated at 300 K, was placed onto the solid substrate surface, allowing it to spread spontaneously. To ensure sufficient spreading and equilibration, the simulation time was set to 6 ns. For the data processing and analysis, atomic trajectory information during the droplet spreading process was recorded, enabling statistical analysis of the relevant properties, such as the spreading radius and contact angle [39].

At the beginning of the simulation, a droplet at 300 K will relax to a thermodynamic equilibrium state and is placed onto the surface of a solid substrate. The droplet spontaneously spreads on the surface of the substrate. To ensure that the droplet fully spreads and reaches equilibrium on the substrate, the simulation time is set to 5 ns. For easier data processing and analysis, the trajectory information of the atoms during the droplet spreading process is recorded. This enables statistical analysis of related information, such as the density distribution, spreading radius, and contact angle.

Each simulation is conducted in regard to the following steps:

1. Initial Setup and Energy Minimization: Water droplets are placed at a distance from the substrate to prevent initial interactions. Excess potential energy in regard to the starting configuration is removed via energy minimization;
2. Equilibration: The droplets are equilibrated at 300 K, with a time step of 1 fs over 0.5 ns;
3. Positioning Near the Substrate: The equilibrated droplets are positioned close to the substrate’s surface;
4. Spreading Simulation: The spreading process is simulated for up to 10 ns to ensure the equilibrium is reached. During this phase:
  - Configurations are recorded every 100 fs for the first 100 ps to capture the initial spreading dynamics;
  - After this stage, the configurations are saved at interval of 1 ps;
5. Data Collection and Analysis: The computational domain is divided into 1 × 1 Å (Y × Z) rectangular bins. The properties in each bin are derived by analyzing the simulation data;
6. Statistical Robustness: For each parameter set, defined by the collision diameter and potential well depth, at least three independent simulations are conducted, each starting from a unique initial state. This minimizes statistical fluctuations and ensures the reliability of the results.

## 3. Results

### 3.1. Spreading Process of a Droplet on Honeycomb-Structured Etched Surface

The variation in the centroid position of a droplet during the spreading process can reflect the spreading rate and serve as an indicator of whether the simulation system has reached the equilibrium. In the initial stage of droplet spreading, the droplet in all cases moves rapidly downward. Since the etched area in the simulation corresponds to a hydrophobic surface, while most areas are hydrophilic, the droplet exhibits a tendency for complete spreading due to strong solid–liquid interactions, forming a stable structure. However, the presence of regularly etched hydrophobic areas introduces significant fluctuations in the spreading process.

Under the influence of an etched hydrophobic surface, the droplet spreading is markedly different from its behavior on a completely smooth hydrophilic surface. The spreading exhibits an oscillatory state, with the diffusion rate displaying a fast–slow–fast pattern that corresponds to the hydrophilic–hydrophobic properties of the surface, and the molecular motion, consequently, becomes more vigorous.

Additionally, variations in the degree of hydrophobicity can further affect the final wettability of the surface.

**Figure 4:**
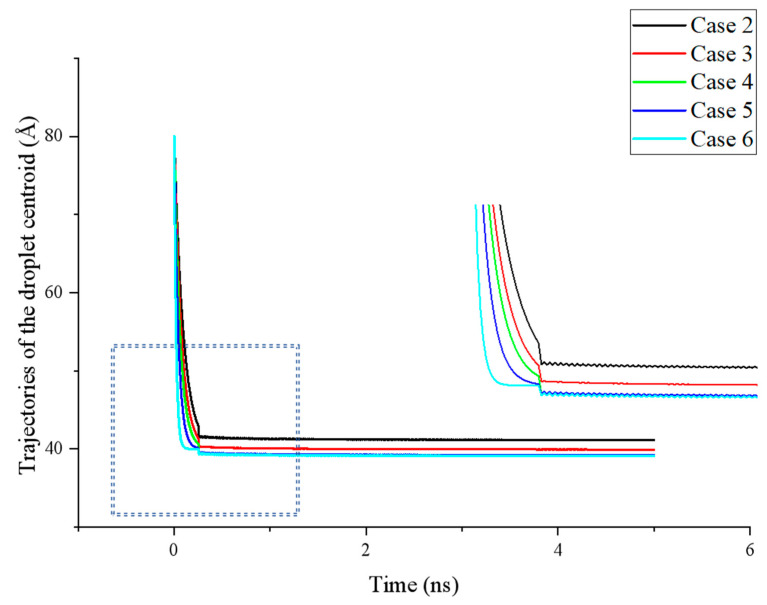
Trajectories of the droplet centroid in all cases.

As shown in Figure 4, during the initial stage of droplet spreading (*t* < 0.5 ns), the droplet in all cases rapidly moves downward. This effect is particularly pronounced when the smooth substrate on the surface exhibits stronger hydrophilicity, allowing the simulation system to reach a dynamic equilibrium state more quickly. Due to the contrasting wettability between the etched and smooth areas of the surface at this stage, the centroid position of the droplet exhibits noticeable fluctuations. The magnitude of these fluctuations increases with greater differences in the wettability. Conversely, when the wettability of the surface areas is more similar (e.g., Case 3 and Case 4), the fluctuations in the droplet’s centroid position are largely reduced.

**Figure 5:**
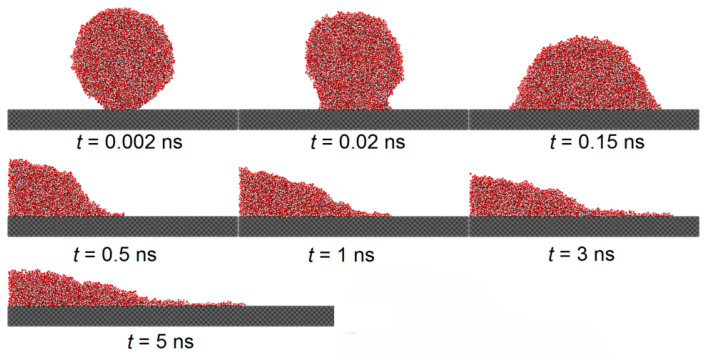
Model identifies spreading phenomenon on a smooth copper surface, from left to right: $\varepsilon_{O-S}=0.9324$.

**Figure 6:**
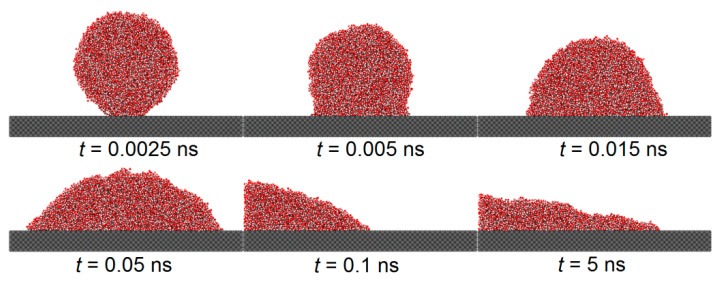
Effects of spreading phenomenon on honeycomb-etched areas, from left to right: $\varepsilon_{O-S}=0.9324$, $\varepsilon_{etched}=0.6216$.

Figure 5 and Figure 6 illustrate the spreading process of a droplet on the substrate surface and the influence of the etched honeycomb structures on droplet spreading. Figure 5 shows the spreading of a droplet on a smooth copper surface, with a solid–liquid interaction of $\varepsilon_{O-S}=0.9324$*,* while Figure 6 depicts the spreading on a similar copper surface, where the hydrophobic areas with a solid–liquid interaction of $\varepsilon_{O-S}=0.6216$ are etched into a honeycomb pattern.

From both the figures, it is found that during the initial phase of droplet spreading, the water molecules near the substrate below the droplet are more easily attracted to the solid atoms and, thus, they move down faster to the substrate surface and form several thick layers of water molecules. At this moment in time, the shape of the droplet is not an ideal spherical crown, and the water molecular layer on the basement surface and the main droplet above it are connected by a sunken “liquid bridge”, which is more obvious when the $\varepsilon_{O-S}$ is large. With the spread of the droplet, the sunken “liquid bridge” gradually disappears, and the main droplet interacts with the water molecular layer on the substrate surface, and there is no obvious distinction between them.

Comparing Figure 5 and Figure 6, it is evident that the etched hydrophobic honeycomb structure reduces the hydrophilicity of the surface, slowing the spreading rate of the droplet and increasing the equilibrium contact angle. The wettability of this etched areas is identified as having a significant role in modulating the overall surface wettability.

### 3.2. Geometric Honeycomb Structure Changes the Wettability of the Surface

The morphological changes of the droplet during the spreading process reflect the differences in the wettability of the substrate. To quantitatively characterize the wettability of the substrate, we tracked the changes in the contact angle and the velocity of the three-phase contact line during the droplet’s spreading process.

**Figure 7:**
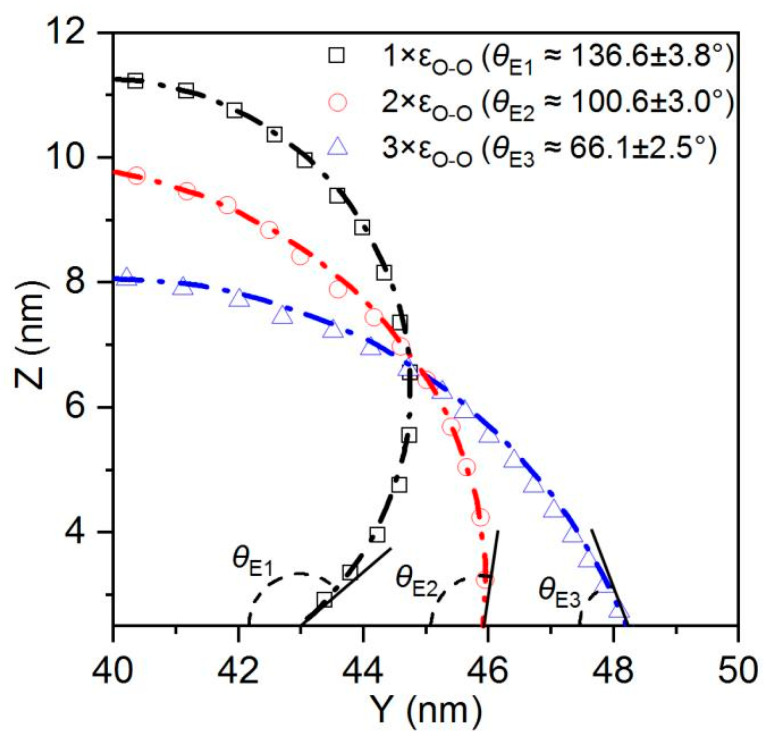
Equilibrium contact angles of the liquid−vapor interface and determination of contact angles in Cases 1, 2, and 3.

**Figure 8:**
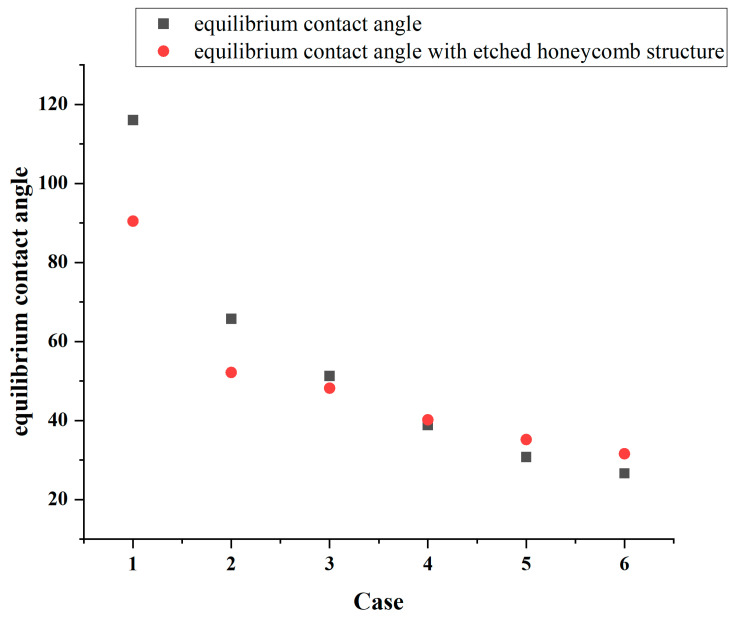
Equilibrium contact angles change after applying the etched honeycomb structure.

As shown in Figure 7, in the absence of etched honeycomb structures, the equilibrium contact angles for Cases 1, 2, and 3 indicate that the smooth copper surfaces in these conditions exhibit a certain degree of hydrophobicity. However, with the addition of more hydrophilic etched honeycomb structures, the surfaces transition from hydrophobic to hydrophilic, as shown in Figure 8.

Figure 8 identifies that the increasing hydrophilicity of the smooth area transforms the etched area from being relatively more hydrophilic to being relatively more hydrophobic, thereby altering the overall surface wettability. In Cases 1, 2, and 3, the equilibrium contact angle of the surface becomes smaller than that of the original, indicating an enhancement in the hydrophilicity. Conversely, in Cases 4, 5, and 6, the etched area is more hydrophobic than the original smooth surface, and the equilibrium contact angle increases, leading to a reduction in the overall hydrophobicity. In Case 1, the original equilibrium contact angle of the surface is 116°, while it decreases by 25.56 to 90.4° after the honeycomb structure is etched onto the surface, while in Case 6, the equilibrium contact angle increases from 26° to 31.51°. The simulation results are shown in Table 3.

Notably, according to Table 3, it is observed that the wettability difference between the etched area and the smooth area is greater, and the variation in the equilibrium contact angle also becomes more pronounced. Since the wettability of the etched area is quantified using an equilibrium contact angle of 45.96°, the etched surface of the honeycomb structure always lies between the wettability of the etched area and that of the smooth area.

This finding demonstrates that etching a honeycomb structure onto the surface and modifying its wettability can effectively alter the overall hydrophilicity or hydrophobicity of the substrate. Creating different wettability effects can serve as an effective strategy for tailoring a surface’s wettability.

### 3.3. Dynamic Analysis of Droplet Spreading on a Honeycomb-Etched Surface

**Figure 9:**
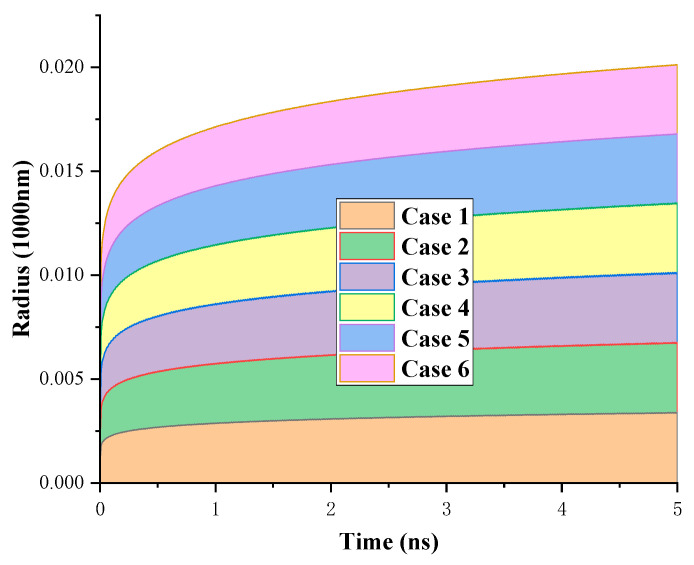
Equilibrium radius of different cases.

As shown in Figure 9, the time evolutions of the droplet spreading radius in different conditions are presented. Overall, the final spreading radius depends on the overall wettability of the surface; the greater the wettability, the larger the final spreading radius.

Furthermore, examining the changes in the dynamic contact angle throughout the spreading process remains necessary to further elucidate the influence of the honeycomb structure on droplet spreading.

**Figure 10:**
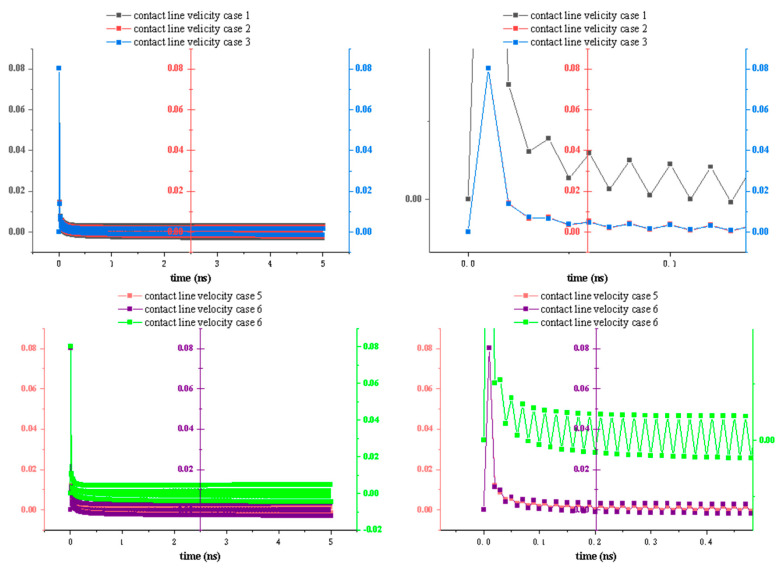
Contact line velocity of different cases.

Therefore, we tracked the droplet’s dynamic contact angle and contact line velocity during the spreading process. Figure 10 illustrates the time evolutions of the dynamic three-phase contact line velocity for Cases 1–6. Based on these profiles, we observe that the droplet initially establishes contact with the surface very rapidly, resulting in a high contact line velocity, which then decreases sharply. According to droplet spreading theory, water molecules first need to establish contact with the surface; subsequently, under the influence of solid–liquid interactions, they spread out across the substrate. During this initial contact phase, the effect of wettability on the contact line velocity is relatively small. However, once contact is established, the droplet’s further spreading behavior becomes closely linked to the substrate’s wettability.

Generating honeycomb-etched patterns influences the contact line velocity, which exhibits noticeable fluctuations due to the periodic honeycomb structures, in contrast to the relatively smooth behavior of droplets on flat surfaces without etching. These fluctuations persist until the droplet reaches the equilibrium and ceases to spread. Moreover, the amplitude of these fluctuations varies across different cases. In particular, Cases 1 and 6 exhibit the largest oscillations in contact line velocity, because the wettability contrast between the smooth and etched areas is most pronounced in these two cases. In the other four cases, as the wettability of the smooth and etched areas becomes increasingly similar, the magnitude of the velocity fluctuations diminishes accordingly.

Because the etched area in this simulation follows a regular honeycomb pattern, we observe that the fluctuations in the contact line velocity correspond closely to the alternating etched and smooth areas. This finding demonstrates that the honeycomb structure indeed influences the motion of water molecules at the microscopic level, impeding their movement in a manner distinct from that of a smooth surface.

## 4. Conclusions and Further Work

### 4.1. Conclusions

In this study, the influence of honeycomb-structured, mixed-wettability surfaces on droplet spreading dynamics and surface wettability modulation was comprehensively investigated through molecular dynamics simulations. The results reveal that a honeycomb structure provides a versatile platform for tailoring surface properties, enabling precise control over the spreading process and equilibrium contact angle. The etched honeycomb areas, with their periodic alternation of hydrophilic and hydrophobic areas, introduce unique oscillatory behaviors during droplet spreading, which are absent on smooth surfaces. These findings underscore the critical role of surface morphology and wettability in determining droplet dynamics at the nanoscale.

The adoption of molecular dynamics simulation enabled the detailed analysis of atomic-level interactions, providing insights that are challenging to achieve through the use of experimental or macroscale numerical approaches. By simulating water droplets on honeycomb-etched surfaces, this study highlights how differences in wettability between etched and smooth areas influence the spreading rate, contact line velocity, and final equilibrium state of the droplet. This work further demonstrates that honeycomb structures can effectively modulate surface wettability, transitioning surfaces from hydrophilic to hydrophobic or vice versa, depending on the design parameters.

In conclusion, this research establishes a strong foundation for the design and application of honeycomb-structured, mixed-wettability surfaces. By enhancing our understanding of nanoscale droplet dynamics and surface interactions, it paves the way for the development of next-generation materials and technologies that leverage the interplay between structure and wettability. Through continued exploration, honeycomb-inspired surfaces hold promise for addressing critical challenges in thermal management, surface engineering, and beyond.

### 4.2. Discussion on Future Work

In the present research, this paper mainly discusses and analyzes water droplet spreading on honeycomb-structured mixed-wettability surfaces through molecular simulation studies. In the future, further experimental validation is required to confirm the accuracy of the model and the applicability of the related surfaces. Although experiments at the nanoscale are currently challenging to achieve, it is planned that the scale of the experimental validation will be extended to the microscale, utilizing surface roughness to create distinct wettability differences between honeycomb-etched and smooth surfaces on certain copper materials to ensure that the experimental conditions are consistent, as much as possible, with the simulation settings. In addition, the droplets should be stably placed through the use of a pipette on the surface. During the spreading process of the droplet, the droplet profile, such as the change in its contact angle and height, needs to be measured in real time. Thus, a comparison of the simulation results and the experimental results can be used to judge whether the simulation model is accurate.

## Figures and Tables

**Table 1:** Non-bond potential parameters of water molecules and copper (Cu) atoms.

Species	Symbol	Mass (g/mol)	q (e)	ε (kcal/mol)	σ (Å)
Hydrogen	H	1.008	0.52	0	0
Oxygen	O	15.9994	−1.04	0.1554	3.1655
Copper	Cu	63.546	0	4.07	2.75

**Table 2:** The 12-6 LJ potential parameters between the water oxygen atoms (O) and solid substrate atoms (S).

Simulation Cases	1	2	3	4	5	6
εo-s (kcal/mol)	0.0154	0.3108	0.4662	0.9324	1.2432	1.154
εo-s (kcal/mol), etched	0.6216	0.6216	0.6216	0.6216	0.6216	0.6216

**Table 3:** Simulation results for the equilibrium contact angles.

Equilibrium Contact Angle	Without Etched	Etched	Difference
90.47	116.03	90.47	−25.56
52.16	65.73	52.16	−13.58
48.16	51.26	48.16	−3.1
40.17	38.81	40.17	1.35
35.15	30.74	35.15	4.41
31.57	26.6	31.57	4.97

## Data Availability

The raw data supporting the conclusions of this article will be made available by the authors on request.
